# Addresses, Etc.

**Published:** 1885-12

**Authors:** P. P. Wells

**Affiliations:** Brooklyn


### ADDRESSES, ETC.

### P. P. Wells, M. D., Brooklyn.

We have had occasion several times in the past few years to express our opinion of the character of some of the "addresses" of presidents of our associated bodies, and not always approvingly. So often have they seemed to be so greatly defective that we have expressed our honest conviction, more than once, that we could better do without them than otherwise, unless we could have them with improved character as to a knowledge of the philosophy of Homœopathy, which treats of the true nature of human sicknesses, and also of that of the agents by which it proposes and promises to cure them. As these continue to come to us, they do not appear to come so improved; and after the last of which we have only an abstract, giving its chief points, we have not been able to avoid the conclusion—If this is the best which the chiefs of Homœopathy have to give us on great occasions, then they, and it, are certainly in a very bad way. Here is one of its utterances as given by the writer of the abstract:

"Then, dealing with the question of attenuations, he made a plea for the use of remedies within that line, wherever it may be, that scientific methods are still able to detect material substance of the drug."

Why this "plea"? What are these "scientific methods"? And what have these "methods" to do with decisions of the best methods and means for the cure of sick humanity? These means, if selected according to law, are addressed to the sensibilities of the living, sick organism, and not to "scientific methods" of any kind, or of methods of any man or men. The object of the administration of any member of our materia medica to any person sick is that he may be cured by it, not that it may be seen by the microscopist, or "detected" by any other "scientific method" whatever. And the only question of its usefulness is—Does it cure? not, can it be seen or "detected"? If *seen*, it is not very apparent how the fact can aid the curing process, or, if "detected" by other means, how the case is the better for this. The objective of our administration of remedies is not for the inspection of the microscopist, or that they may be brought to test by any, whatever, of "scientific methods." If the means given to the sick for their cure accomplish this object, or if they fail to do this, they have been subjected to the *only* "scientific method" which can have any possible "*scientific*" relation to the case. The whole problem of sickness and cure is in the domain of dynamics, and never at all in that of physics. And, hence, any reference here to processes which are applicable to investigations in physics is about as congruous and logical as to propose resort to the methods and means of the chemist's laboratory for investigations into the philosophy of morals. The "plea" in this case is too evidently only the outcome of ignorance of the real nature of the factors of the problem he was attempting to discuss. It would be better if such ignorance hereafter could be counted a disqualifying element in choosing presidents for so important bodies as our State societies.

And then—

"But if he were alive to-day [*i. e.*, Hahnemann], and having kept astride [whatever this may be] with the times in the various departments of medicine, his logical mind would ere this have given to his discovery its proper *limited* place."

How *limited?* and by whom or by what? This "logical mind," which this President no doubt means to compliment, supposed his "*discovery*" was a *natural law*. What shall be thought of the intelligence of a President who talks of "*limiting*" such laws—that of gravitation, for example? Why this, which underlies the science of therapeutics, more than that? The *universality* of its applicability to the cure of all curable human sicknesses is one of the irrefragable proofs of its origin and nature—that it is indeed a God-given law, and, like other laws having the same origin, has no "*limits*." Is this talk of "*limits*" to the operations of a natural law another evidence that ignorance of the subject he was professing to discuss disqualified him for his office and for the discussion this imposed on him? It may be, and it may be evidence of this will increase as we progress. And then it may be, if Hahnemann could only have lived and "kept astride with the times," something wonderful might have happened. But as he didn't live and he didn't fix a limit to the operation of the natural law he discovered, it is somewhat appalling to contemplate the power and extent of a "stride" which could be attended with such consequences. And then, given the "stride," how did this President know such consequences would follow? We do not see how he could know—and the more, as the evidence in this abstract of his address is ample that he is neither a prophet nor the son of a prophet.

"He then spoke of the great superiority of homœopathic medication in pneumonia over the old methods of allopathic prescribing, but contrasted with the modern expectant method its superiority was shown by statistics to be only 1.7 per cent."

We take issue with this alleged testimony of statistics as to the comparative results of expectant and homœopathic treatment of pneumonia. The President expresses near enough the comparative results of Fleischman's treatment of this disease in his hospital in the Gumpendorf suburb of Vienna with the expectant treatment of the disease as given by Dietl. But Fleischman was never a representative homœopathic prescriber; and in the cases reported in the *Œsterreiche Zeitschrift fur Homœopathie*, Band I, Heft 1, S. 198, his practice was *not homœopathic*. It is described by himself in the *Zeitschrift*, at the page referred to, as only treated by Phosphorus from the beginning to the end. He sustains this practice as follows: "The experience of many years has given me the strongest conviction that it [pneumonia] can be cured by no other remedy so safely and speedily as by Phosphorus, without the help of any other remedy." This certainly is not *homœopathic* treatment. It was only treatment based on a *name*, not on the *totality of the symptoms*, without which no treatment is homœopathic.

The proper homœopathic treatment of pneumonia will show a result of loss much less than 6 5 per cent., which was Fleischman's loss under his Phosphorus treatment. He subsequently changed his method and reduced his mortality to $3\frac{31}{75}$ per cent., or nearly one-half.* And even this, we believe, does not fairly represent the loss from pneumonia under a right homœopathic treatment. We believe this, because we have the proof of this in our own experience. In a practice of this system which reaches back forty-three and two-thirds years, which most of the time has been very large, and of a general character as to the diseases treated, of which, no doubt, pneumonia has made an average part, I have *not lost one case,* and we are sure this is better than the difference between 6.5 and 7.4, from which probably our President derived his 1.7 per cent., or the difference between Homœopathy and Dietl's expectant experiment. Muhlenbein, in a homœopathic practice of twenty-seven years, lost less than one per cent. in all these years (one in one hundred and five). His was a general practice, and, of course, he treated his share of pneumonia. And then he, the President,

"desired the Society [the New York State] to lay aside as much of the mysterious as possible and give prescriptions that contain *matter*, thus being more likely to obtain force by its administration and obtain the best possible results in the treatment of disease."

---

* Reis, in hospital in Linz, in a practice from 1843 to 1848, lost one per cent., so that, presumably, of every 6.5 lost by Fleischman under his *quasi* homœopathic treatment, 5 5 would have been saved if they had been really treated homœopathically; and of the 7.4 per cent. who died under the expectant treatment of Dietl, 6.4 might have been saved. The difference of loss between true homœopathic treatment and the expectant is greater by many times than 1.7 per cent—it is 6.4 saved out of every 7.4 lost, or six hundred and forty saved from seven hundred and forty lost.

Then the duration of cases under Dietl's expectant treatment averaged three weeks, or twenty-one days, while under homœopathic treatment, with the thirtieth number, the average was but 11.3 days; treated with the fifteenth, 14.6 days; by the sixth, in which "*matter*" is admitted to be present in the doses, the duration was 19.5 days, differing but little from the result of the expectant treatment. These facts, which were gathered from a series of experiments in the homœopathic hospital in the Leopold-Stadt, in Vienna, under Wurmb, are worthy of the careful attention of such as our President, who will have *matter* in their doses. They will find that in the proportion as the doses approached the point of alleged *visible matter* the result came nearer to that of the expectant or no treatment at all. They will not fail to notice that under treatment by the highest number used the duration was but little more than half that where there was *matter* in the dose.

This is certainly as precious a specimen of *begging the question* as we remember to have seen, and this the world has adjudged as of no great force in argument. Give "*matter*" and so get "more force," and so "the best possible results in the treatment of disease." To what force does this apparently very ignorant President refer? Is it to the "force" which cures or the "force" which poisons—kills? If to the former, then, before he allowed himself to be put into the President's chair he ought to have known that the best possible testimony to be had in the case declares that this curing power is greater in doses where there is no evidence of matter than in those where presumably matter may be found. These witnesses say this not because of any speculative reasoning on the subject, but because they have tried it and found it to be a fact; and having so proved the fact, it must be accepted as against the dictum of our President, who evidently knows nothing at all about it. And then, if he would lay aside the "mysterious" in practical medicine, he must discard all of knowledge of life—sick and in health—all of the agents by which sick life is to be restored. All here is mystery. But if he means "lay aside" all affectation of mystery on the part of the prescriber, he is right and we are with him.

"The next portion of the address was a warning with regard to certain influences threatening to destroy Homœopathy as legalized in the State of New York, in the shape of associations composed of homœopathic physicians and antagonistic to the State Society."

This warning may have been very sincere, and the apparent danger may have given our President great pain. It is, therefore, a pleasure to assure him that if his fears are for the safety of Homœopathy, or of aught of good belonging to it, he need have no fear for either, for all this will live and grow, even if the antagonism deplored should succeed in extinguishing the State Society; and it is not quite sure these would not do nearly as well without the State Society as with it. If the fear is for the safe continuance of the State Society, he may be reminded that this was declared to have committed "hara-kari" some years ago—*verdict*, died by its own hand—and this by reason of a certain "resolution" which was made to *pass through its body*. If it has in any measure revived by reason of large doses of material philosophy it has been enduring, it is not very apparent that it is restored to any sense or knowledge of that of Hahnemann. This has hardly left a trace on the subsequent record of the organization. And then we would gladly assure him we have no thought these organizations to which he alludes have any malign intentions or wishes as to that over which he presided. It may well be that they have no great relish for the large doses of material philosophy with which his Society has been drugged, and I can easily see how, if they have any such destructive wishes as he intimates they have, they have only to await the natural results of these large doses, and that now partly accomplished, in this greater organization—viz.: the near death of all which is characteristic of true Homœopathy, and, consequently, the extinction of the only *raison d'être* the State Society ever had. Its consequent "*second*" death will be quite *natural.* And then as to these associations. It is not quite clear what the President and his Society can do with them other than hate them, and this it is evident they can do. Notwithstanding this, we would assure him these homœopathic physicians who constitute these associations, so warned against, are a very respectable and intelligent body of men, and are perfectly harmless as to all except the enemies of true Homœopathy, and we would advise all such, societies or men, to "*give them a wide berth.*"

And further, for the instruction of our President and his Society, we have been told, and we see no reason to doubt the statement, that at least one of the "associations" probably alluded to had its origin in the fact that our State Society had so little to do with Hahnemann's Homœopathy. This being so, then it may be there is something our State Society can do. Will they do it? Or will they rather heed the suggestion of the President, said to have been given in these words:

> "Attention was next called to the limited instruction given students in our colleges in materia medica, and the importance of familiarizing them with the materia medica of the old school."

Why is this important? What is there now in old school materia medica, which is not in the new, which can be of the least importance to a student of Homœopathy, or to a practitioner of this, who has been a *student* of its materia medica, in any discharge of his practical duties? There is in it really nothing, except what has been taken from the new, and this can be better studied in its original connection by any one who is to practice curing the sick under the guidance of law. We cannot do otherwise with this suggestion than to refer it to the limbo of the silly and the useless, with that other, not more silly, which would incorporate into a homœopathic curriculum a knowledge of how to prepare pukes, purges, and poultices. The student and practitioner of Homœopathy has no use for either, and the suggestion of either of these to such, as of possible helps in clinical duties, can only have had origin in the densest ignorance of all belonging to the dynamic system of practical medicine taught by Hahnemann. This system has resources of its own so ample and so superior to all these, that a need of any of them can never be felt by one who has given intelligent and honest study to homœopathic law and the means it presents for the cure of the sick. Such suggestions can only have come from a sickly sense of imbecility to deal with a law not understood, and with its methods, which, to these suggestors, are but strangers.

The abstracts of the address on which we have commented are taken from the first number of the first volume of the new series of the *North American Journal of Homœopathy*, which has come to us with bright outlook and promise. A brief editorial in this number, evidently having this address in view, says:

"On what line is true progress in the homœopathic school to move? Upon the line of appreciable matter, preconceived as the limit of the dose, or upon the line of induction from scientific and unbiased clinical experiments?"

The reply to this question must depend very much on answers to other questions, not here put by the respected editor: 1st. Is the progress to be upward and onward, or downward and backward? If the example and record of the greatest and best of the masters of homœopathic science and prescribing are to be our instructors, we are answered as to the "line' on which "true progress" *has* moved, and if this example and record are compared in their results with any differing from these, we shall have no difficulty in seeing on what "line" "true progress" *must* "move," and very little knowledge of the history of this "progress" will demonstrate that it can move on no other. There have not been wanting, nor are there wanting now, efforts to "move" on "lines" differing from that pursued by these worthies, but the "progress" on these has always been and is now away from the truth, downward and backward into a near approach to the murk and groping of allopathy. These downward and backward movements and movers have always been in sympathy with this, and seem not seldom to have been inspired by desire and hope for an ultimate "union" of Homœopathy with this, "on a *scientific* basis." That is to say, "movements" on this other "line" have for their objective the final extinction of Homœopathy. Homœopathy in its nature and in the means it employs is *dynamic*. These other movements and movers will only have to do with *matter*—matter in their doses—matter which can be *seen*, and they will have no other. Just so with old school physic. And is a progress of Homœopathy toward this in any sense or degree a "*true* progress"?

The first of the worthies to be named by whose example and experience we should be instructed is Hahnemann himself. He "moved" on the "true" "line" from the initiation of his discovery of the law of therapeutics to the development of means which could be most *safely* used for the cure of the sick, and with least addition to their sufferings. This was his only motive when he entered on the experiments which revealed to him the fundamental principle in homœopathic dynamization. He sought to avoid aggravation of sufferings from excessive doses, and only this. He found the reduction of the *matter* of the dose did not diminish, but rather increased the *curing power*. He "moved" on this "line" of reduction till he reached the thirtieth in the centesimal series, and here, having gained his objective, escape of aggravations, he stopped. Why he did not go on with his discovery of increase of *curing power* when *matter* was decreased, is not quite apparent.

The next "move" was made by Korsakoff. His object was to find the limit of the curing power in the advancing series, the idea being that the increased power heretofore realized was only the increased division of the molecular constitution of the drug, and this must somewhere cease to be further divided, and then the curing power would cease altogether. With this end in view he carried Sulphur and some other anti-psorics, phial by phial, to the fifteen hundredth number. He cured an obstinate chronic affection with this number and was surprised. He thought he must have passed the limit of curing numbers long before this. Here he gave up his search for the limit. On this "line" on which he "moved" he had made "progress" in knowledge of the nature of the curing power, and as this was the result of intelligent experiment and observation the "progress" must have been a "true" one. It demonstrated the fact that *that* which cured was not the *matter* of the drug. For here the power cured, where, if it were the *matter* of the drug, it had failed when more of it had been given. Shall we be instructed by Hahnemann and Korsakoff?

On the same "line" comes next after Korsakoff, Jenichen, who moved as far as the forty thousandth in the centesimal series, in the case of Arsenicum, and thirteen thousandth of Phosphorus. His high numbers were taken up by Stapf, the elder Gross, Bœnninghausen, Hering, and many others, who had most interesting experiences and successes as the result of their use. Even in his highest number of Arsenic they found not only had the limit of its curing power not been passed, but that even here it was present in great power. I have myself seen the discharging, open cancer, in its most painful and offensive form, altogether changed as to pains, soreness, foulness, and quantity of the discharge by this highest number of Jenichen. The constitutional irritability and fever were also greatly reduced after its use. There was no mistaking the beneficent action of the medicine. This was so apparent that my enemy and enemy of Homœopathy had sufficient influence to cause the removal of the patient to a distant city where she would be out of my way and that of "high potencies!"

Is it not apparent that Jenichen "moved" on the right "line"—and the more when we hear those truly great masters of our art declare they found a greater curing power in these higher numbers than they had ever met in the lower? Gross exclaims, after witnessing the result of his first prescription of Jenichen's high numbers, "*Wie gross war meine erstaunen!*" [How great was my astonishment!] Who can be so foolish as not to recognize this "move" of Jenichen as on the right "line," and who so stupid as to refuse to be instructed by the example and experiences of these truly great masters? And the more when past history proves that no "true progress" in therapeutics has been made on any other "line," and past experience and the nature of the case equally prove that "true progress" in therapeutics is possible on no other—certainly not on the material "line"? Progress on the dynamic "line" is only a carrying out of Hahnemann's discovery toward its logical and natural termination.

